# The PKA-C3 catalytic subunit is required in two pairs of interneurons for successful mating of Drosophila

**DOI:** 10.1038/s41598-018-20697-3

**Published:** 2018-02-06

**Authors:** Marlène Cassar, Elizabeth Sunderhaus, Jill S. Wentzell, Sara Kuntz, Roland Strauss, Doris Kretzschmar

**Affiliations:** 1 Oregon Institute of Occupational Health Sciences, Oregon Health & Sciences University, Portland, OR 97239 USA; 2 Institut für Entwicklungsbiologie und Neurobiologie, Universität Mainz, Colonel-Kleinmann-Weg 2, 55099 Mainz, Germany; 3 Present Address: Department of Biology, Duke University, Durham, NC 27708 USA

## Abstract

Protein kinase A (PKA) has been shown to play a role in a plethora of cellular processes ranging from development to memory formation. Its activity is mediated by the catalytic subunits whereby many species express several paralogs. *Drosophila* encodes three catalytic subunits (PKA-C1–3) and whereas PKA-C1 has been well studied, the functions of the other two subunits were unknown. PKA-C3 is the orthologue of mammalian PRKX/Pkare and they are structurally more closely related to each other than to other catalytic subunits within their species. PRKX is expressed in the nervous system in mice but its function is also unknown. We now show that the loss of PKA-C3 in *Drosophila* causes copulation defects, though the flies are active and show no defects in other courtship behaviours. This phenotype is specifically due to the loss of PKA-C3 because PKA-C1 cannot replace PKA-C3. PKA-C3 is expressed in two pairs of interneurons that send projections to the ventro-lateral protocerebrum and the mushroom bodies and that synapse onto motor neurons in the ventral nerve cord. Rescue experiments show that expression of PKA-C3 in these interneurons is sufficient for copulation, suggesting a role in relaying information from the sensory system to motor neurons to initiate copulation.

## Introduction

Protein kinase A (PKA) is a key regulator in many processes, including cellular growth, embryonic patterning, and learning and memory formation in flies and mammals^1–6^. PKA has also been connected with sexual behaviour because PKA activity increases when male mice interact with females and mutations in the PKA-regulatory subunit impair courtship suppression in *Drosophila*, which occurs after males encounter already mated females^7–10^. PKA is a tetramer of two regulatory and two catalytic subunits, whereby several paralogs for these subunits are found in mammals^11^ as well as *Drosophila*. Flies encode three catalytic subunits, PKA-C1–3^12,13^, but functional studies have only been performed with PKA-C1 and the functions of PKA-C3 were unknown. *Pka-C3* transcripts have been detected in Northern blot analyses, which revealed expression in pupae and adult heads while weak expression was found in embryos and larvae^13^. To address a functional redundancy with PKA-C1, PKA-C3 was induced in PKA-C1 mutants by fusing its coding region to the PKA-C1 promoter, however, this did not rescue the phenotypes caused by the loss of PKA-C1^14^. This suggested that the different catalytic subunits have specific functions that cannot be fulfilled by other subtypes. PKA-C3 is evolutionary highly conserved and interestingly it is structurally more closely related to its mammalian orthologue PRKX (also called Pkare) than to PKA-C1 or PKA-C2^15–17^. PRKX is expressed in the developing and adult mouse brain, whereby the pattern during development is restricted to differentiating neurons in the first ganglion, the dorsal root ganglia, and the mantle layer of the telencephalon^15,18^. PRKX is also found in non-neuronal tissues, such as testes and kidney, and knocking it down in kidney explants resulted in decreased ureteric bud branching^19,20^. In contrast, neuronal functions of PRKX are also still unknown. We previously found that PKA-C3 activity is inhibited by Swiss-Cheese (SWS) which acts as a non-canonical regulatory subunit that binds to PKA-C3, tethers it to membranes, and inhibits its activity^21^. SWS specifically interacts with PKA-C3 and does not bind to PKA-C1 or PKA-C2, again supporting unique roles of this unusual PKA complex. However, what these roles are and what consequences the loss of PKA-C3 has for neuronal integrity and function remained unknown.

## Results

### Loss of PKA-C3 does not affect brain development or integrity

To identify functions of PKA-C3, we first knocked it down pan-neuronally using the *Pka-C3*^*NIG*.*6117R*^ RNAi construct and the *Appl*-GAL4 driver line. The knockdown was confirmed by performing RT-qPCRs from head homogenates (Supp. Figure 1). The knockdown flies were viable and did not show any overt defects. To determine whether brain development or survival was affected, we performed paraffin head sections of 3d and 30d old flies but neither revealed detectable changes compared to age-matched controls (data not shown). This suggested that the loss of PKA-C3 does not interfere with the development of the brain or with the maintenance of brain integrity during aging. To confirm this, we generated a mutation in PKA-C3. Because no classical allele was available, we created a deletion in the *Pka-C3* gene using two piggyBac insertions and FLP/FRT-mediated recombination^22^. Two alternative transcripts, RA and RB, using different first exons are predicted to be transcribed from the *PKA-C3* locus^23^ and the deletion removes all coding exons of the RA transcript and all coding exons besides the first for RB (Supp. Figure 2A). The first exon of RB encodes 13 amino acids which do not contribute to any known functional domain. The deletion was confirmed by PCR (data not shown) and Western blots that verified that no PKA-C3 protein was detectable in the deletion line (Supp. Figure 2B). Using this mutant, called *Pka-C3*^*d*^, confirmed that the loss of PKA-C3 did not cause defects in the development of the brain or its maintenance during aging (data not shown).

### PKA-C3 is required for copulating

**Figure 1:**
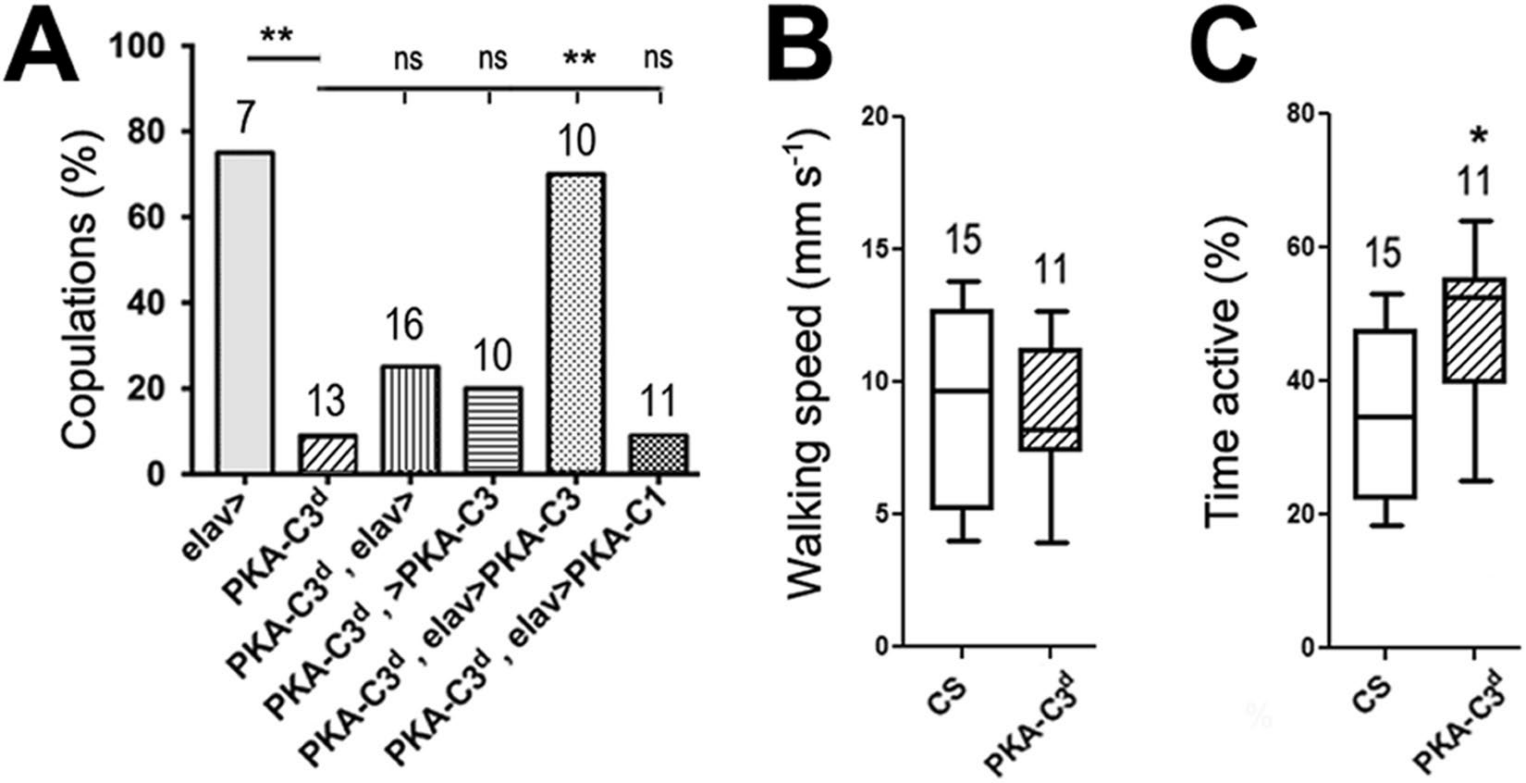
*Pka-C3*^*d*^ mutant males show copulation defects. (**A**) Percentage of males that copulated with wild-type CS females within the observation period of 30 min. *Pka-C3*^*d*^ males performed significantly worse than controls (*elav*-GAL4). Inducing PKA-C3 with *elav*-GAL4 restored mating behaviour of *Pka-C3*^*d*^ males whereas carrying only the *elav*-GAL4 driver or the UAS-*PKA-C3* construct had no significant effect (ns). Expressing PKA-C1 in *Pka-C3*^*d*^ could also not rescue the copulation defects. Flies were 3d old. (**B**) Walking speed, measured in the Buridan’s paradigm, was not affected in 14d old *PKA-C3*^*d*^ mutants compared to wild-type CS. (**C**) *Pka-C3*^*d*^ mutants were more active than wild-type CS controls in the Buridan’s paradigm during the 15-min observation period. Number of individual flies tested is indicated on each bar. A Chi-square test was used to determine significance in A and unpaired student t-tests were used to determine significance in B and C. (**B**,**C**) Horizontal lines are medians; boxes are 25 and 75% quartiles; and whiskers, are 10 and 90% quantiles. *p < 0.05, **p < 0.01.

In the creation of *Pka-C3*^*d*^, we noticed that the flies were sterile when homozygous. Whereas *Pka-C3*^*d*^ females produced progeny when crossed to wild-type males, this was not the case when we used *Pka-C3*^*d*^ males and wild-type females. Determining the courtship behaviour of *Pka-C3*^*d*^ males by measuring the time they engaged in stereotypical courtship behaviours, like chasing the wild-type females or extending their wings, we failed to detect a significant difference to wild-type males. (Supp. Figure 3). However, only 9% of the *Pka-C3*^*d*^ males attempted to copulate with the females within a 30 min period compared to 71% of the control males (Fig. 1A), showing that the loss of PKA-C3 specifically impaired this component of courtship. Next, we determined whether the copulation deficits are specifically due to the loss of PKA-C3 by performing rescue experiments. As expected, pan-neuronal expression of PKA-C3 via *elav*-GAL4 in *Pka-C3*^*d*^ mutants restored the copulation frequency (Fig. 1A). In contrast, inducing PKA-C1 did not increase copulations and these flies were not significantly different from the *PKA-C3*^*d*^ mutant flies (Fig. 1A). This reveals that PKA-C3 has specific functions that cannot be compensated by PKA-C1, although they show strong conservation in their catalytic domain (Supp. Figure 4). In addition, it further supports that PKA-C3 and its mammalian orthologues may form a unique subclass of PKA catalytic subunits with specific functions.

Because the mating defects could be due to reduced activity or locomotion, we measured activity and walking speed of *Pka-C3*^*d*^ flies in the Buridan’s paradigm^24^. In this assay, the flies are recorded while walking back and forth between two inaccessible landmarks and the recordings of this behaviour were used to analyse walking speed and general activity. Analysing the walking speed of *Pka-C3*^*d*^ flies demonstrated that they were as fast as wild-type flies (Fig. 1B) and quantifying the time the flies were active showed that *Pka-C3*^*d*^ flies actually spend more time moving around than wild-type (Fig. 1C). Together, this shows that the mutant flies are not impaired in their locomotion nor are they inactive. Furthermore, the flies consistently walking back and forth between the landmarks suggested that the loss of PKA-C3 did not interfere with their vision. These findings indicate that the defects in mating are due to deficits in integrating information to orchestrate the appropriate behavioural output, rather than defects in sensory input or motor neuron output.

### PKA-C3 is expressed in the ADLI and ICLI interneurons

**Figure 2:**
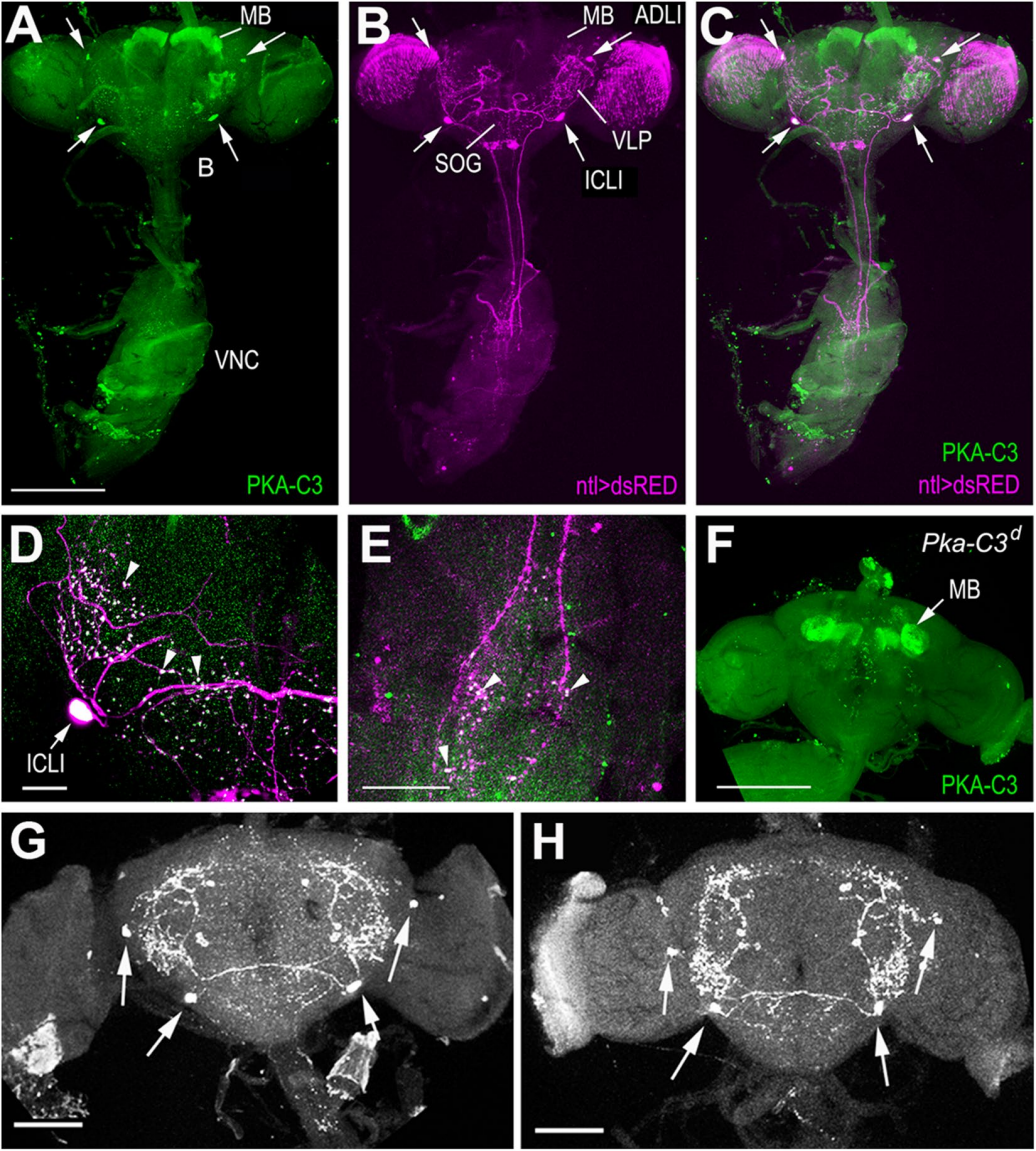
PKA-C3 is expressed in the ADLI and ICLI interneurons. (**A**) Immunohistochemistry on brain/ventral nerve cord preparations using our anti-PKA-C3 antiserum. Strong PKA-C3-positive staining is detectable in the mushroom bodies (MB) and two pairs of neurons in the protocerebrum (arrows). (**B**) Expressing DsRed with *ntl*-GAL4 shows staining in the ADLI (anterior dorso-lateral) and ICLI (inferior contralateral) interneurons that send projections from the brain (B) to the ventral nerve cord (VNC). (**C**) Overlay of the PKA-C3 and DsRed staining shows expression of PKA-C3 in the ADLIs and ICLIs (arrows). (**D**) Magnification showing one of the ICLI neurons reveals PKA-C3 positive staining in the cell body (arrow) and the varicosities formed by the ICLI neurites (arrowheads). (**E**) PKA-C3 can also be detected in the varicosities of the ICLIs in the VNC (arrowheads). (**F**) Immunohistochemistry with anti-PKA-C3 on a brain whole-mount from a *Pka-C3*^*d*^ mutant shows unspecific staining in the MBs whereas the ADLIs and ICLIs are not detectable. (**G**,**H**) Loss of PKA-C3 does not interfere with ADLI and ICLI survival because we can detect them in wild-type flies (**G**) as well as in *PKA-C3*^*d*^ flies (**H**) using an antiserum against Natalisin. The arrows point to the cell bodies of the ADLIs and ICLIs. SOG; = suboesophageal ganglion, VLP; = ventro-lateral protocerebrum. Scale bar in A, F = 200 µm, in D, E = 50 µm, in G, H = 100 µm.

Our pan-neuronal rescue experiments showed that PKA-C3 is required in neurons. To identify whether PKA-C3 is needed in specific neuronal subtypes, we performed immunohistochemistry to determine PKA-C3’s expression pattern with an antibody raised against PKA-C3 in chicken (using the DTKNFDDYPEKDWKPAK peptide highlighted in Supp. Figure 4). Using this antibody on brain/ventral nerve cord preparations, we found strong staining in the mushroom bodies (MB) and in a few large neurons in the protocerebrum (arrows, Fig. 2A). To identify these neurons, we expressed DsRed with various GAL4 lines and performed immunohistochemistry using anti-DsRed and anti-PKA-C3. As shown in Fig. 2C, we found a co-localization of PKA-C3 and DsRed when using *natalisin* (*ntl*)-GAL4. Natalisin is a tachykinin-related peptide that is expressed in two pairs of interneurons, the anterior dorso-lateral interneurons (ADLIs) and the inferior contralateral interneurons (ICLIs)^25^. Furthermore, *natalisin* has been shown to be involved in mating behaviour, however in contrast to PKA-C3 in both, males and females^25^. The ADLIs and ICLIs form an extensive neuritic network in the brain that connects the ventro-lateral protocerebrum (VLP), the anterior suboesophageal ganglion (SOG) and the MBs and they send axons into the ventral nerve cord (Fig. 2B and Supp. Figure 5)^25^. In the brain as well as in the ventral nerve cord, they form varicosities that are positive for Natalisin and interestingly PKA-C3 also predominantly localizes to these varicosities (arrowheads, Fig. 2D,E). To confirm that the immunostaining in these neurons was specific for PKA-C3, we used brain/ventral nerve cord preparations from *Pka-C3*^*d*^ mutants. In these preparations the ICLIs and ADLIs were not stained, however the staining of the mushroom bodies persisted (Fig. 2F). A similar result was obtained when using *Appl*-GAL4 induced knockdown PKA-C3 flies which showed a dramatic reduction of PKA-C3 in the ICLIs and ADLIs while the mushroom bodies were still labelled (Supp. Fig. 6). We therefore concluded that the staining in the MBs is due to cross-reactivity of the antibody with another antigen. To address whether the loss of PKA-C3 positive staining could be due to a loss of the ADLIs and ICLIs in *Pka-C3*^*d*^ mutants, we used an antiserum against Natalisin. Because we could easily detect the ADLIs and ICLIs in wild type (Fig. 2G) as well as in *Pka-C3*^*d*^ (Fig. 2H) with this antiserum, the loss of PKA-C3 staining was not due to the loss of these cells but to the loss of PKA-C3 in these neurons. This was also confirmed by expressing GFP with *ntl*-GAL4 in *Pka-C3*^*d*^ mutants (data not shown).

### Loss of PKA-C3 in the ADLIs and ICLIs is sufficient to restore copulation

**Figure 3:**
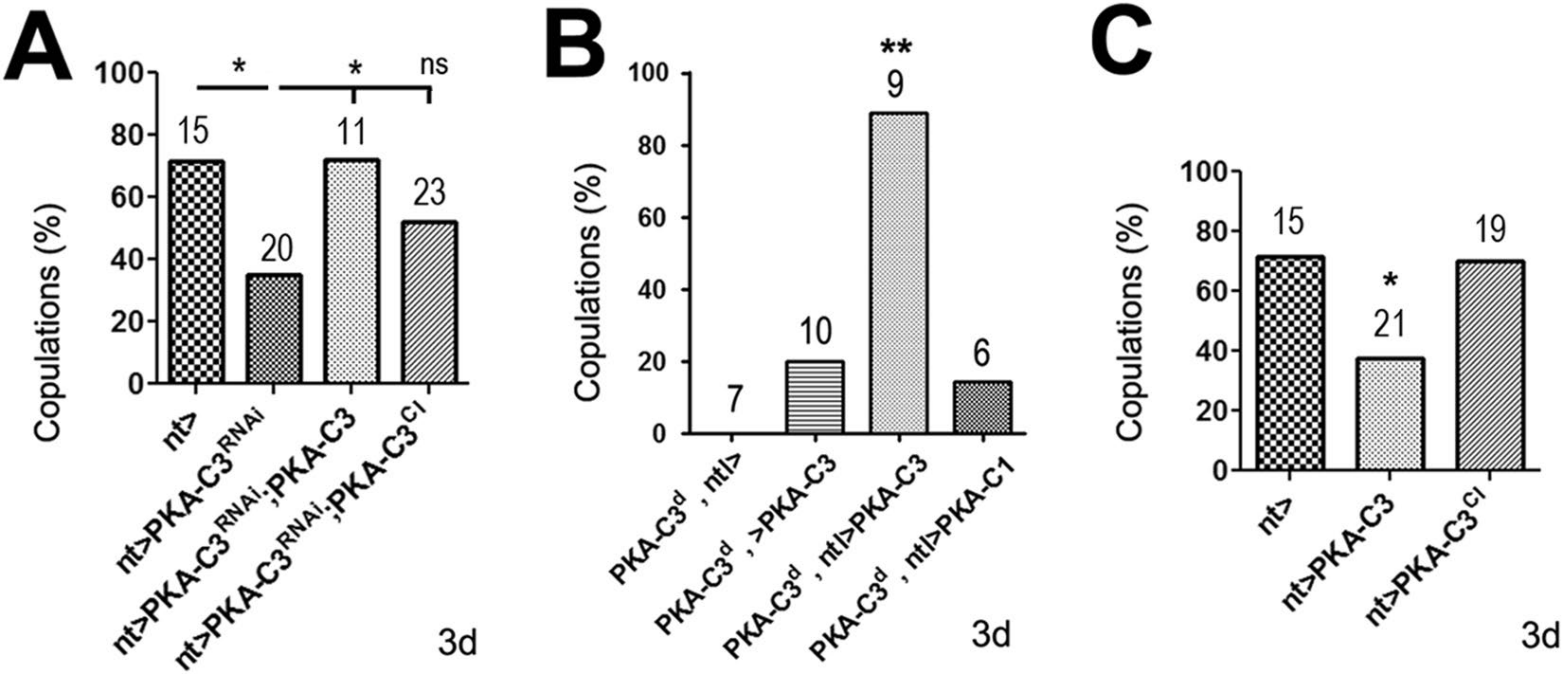
Expression of PKA-C3 is required in the ADLIs and ICLIs. (**A**) Knocking down PKA-C3 with *ntl*-GAL4 and PKA-C3^NIG.6117R^ causes reduces copulations of 3old males. Expressing wild-type PKA-C3 with *ntl*-GAL4 in the knockdown flies restores copulations whereas expression of the constitutively inactive PKA-C3 (PKA-C3^CI^) does not. (**B**) Expressing PKA-C3 with *ntl*-GAL4 in *Pka-C3*^*d*^ mutants is sufficient to restore mating while expression of PKA-C1 has no effect. (**C**) *ntl*-GAL4 driven expression of PKA-C3 in the wild-type background reduces copulations while additional expression of PKA-C3^CI^ does not. Statistical analysis was done with one way Anova and Chi-square tests. Number of individuals tested and the SEMs are indicated. *p < 0.05, **p < 0.01, ns not significant.

The immunohistochemistry reveals that PKA-C3 is expressed in the ADLI and ICLI interneurons and due to their morphology, these neurons could play an important role in integrating information that then controls behavioural output. We therefore first addressed whether the loss of PKA-C3 in these neurons is sufficient to induce the copulation deficits by expressing the Pka-C3^NIG.6117R^ RNAi construct with the *ntl*-GAL4 driver. Analyzing courtship behaviour, we found a significant reduction in copulation events when *ntl*-GAL4 induced PKA-C3 knockdown males were presented with wild-type females (Fig. 3A). This confirms that PKA-C3 is necessary in these two pairs of neurons for normal mating. Next, we investigated whether expression of PKA-C3 in the ADLIs and ICLIs is sufficient to rescue the phenotypes after the loss of PKA-C3. As shown in Fig. 3A,B, induction of PKA-C3 via *ntl*-GAL4 was sufficient to restore mating behaviour of PKA-C3 knockdown mutant males. We also addressed whether the catalytic activity of PKA-C3 is required for its function by using a constitutively inactive form of PKA-C3 in which aspartate 397 in the catalytic loop is replaced by alanine (see Supp. Figure 3). Although this construct is expressed at higher levels than our normal PKA-C3 construct, it did not increase PKA activity whereas PKA-C3 did (Supp. Figure 7), suggesting that it is indeed a catalytically inactive form. In contrast to PKA-C3, the inactive PKA-C3^D397A^ did not rescue the copulation defects (Fig. 3A), showing that the PKA catalytic activity is required for the function of PKA-C3. As expected, expression of PKA-C3 with *ntl*-GAL4 also restored the mating behaviour of *Pka-C3*^*d*^ mutant males. As shown with the pan-neuronal expression, induction of PKA-C1 with *ntl*-GAL4 did not rescue the mating defects (Fig. 3B), again confirming the specificity of PKA-C3. Lastly, we tested whether overexpression of PKA-C3 in Natalisn neurons induces mating phenotypes. As shown in Fig. 3C, induction of wild-type PKA-C3 did reduce the copulation events, whereas the inactive form did not. This suggests that the levels of PKA-C3 have to be tightly regulated and that the catalytic activity is required for both, restoring the normal function and causing overexpression phenotypes.

### The ADLIs and ICLIs connect onto motor neurons

**Figure 4:**
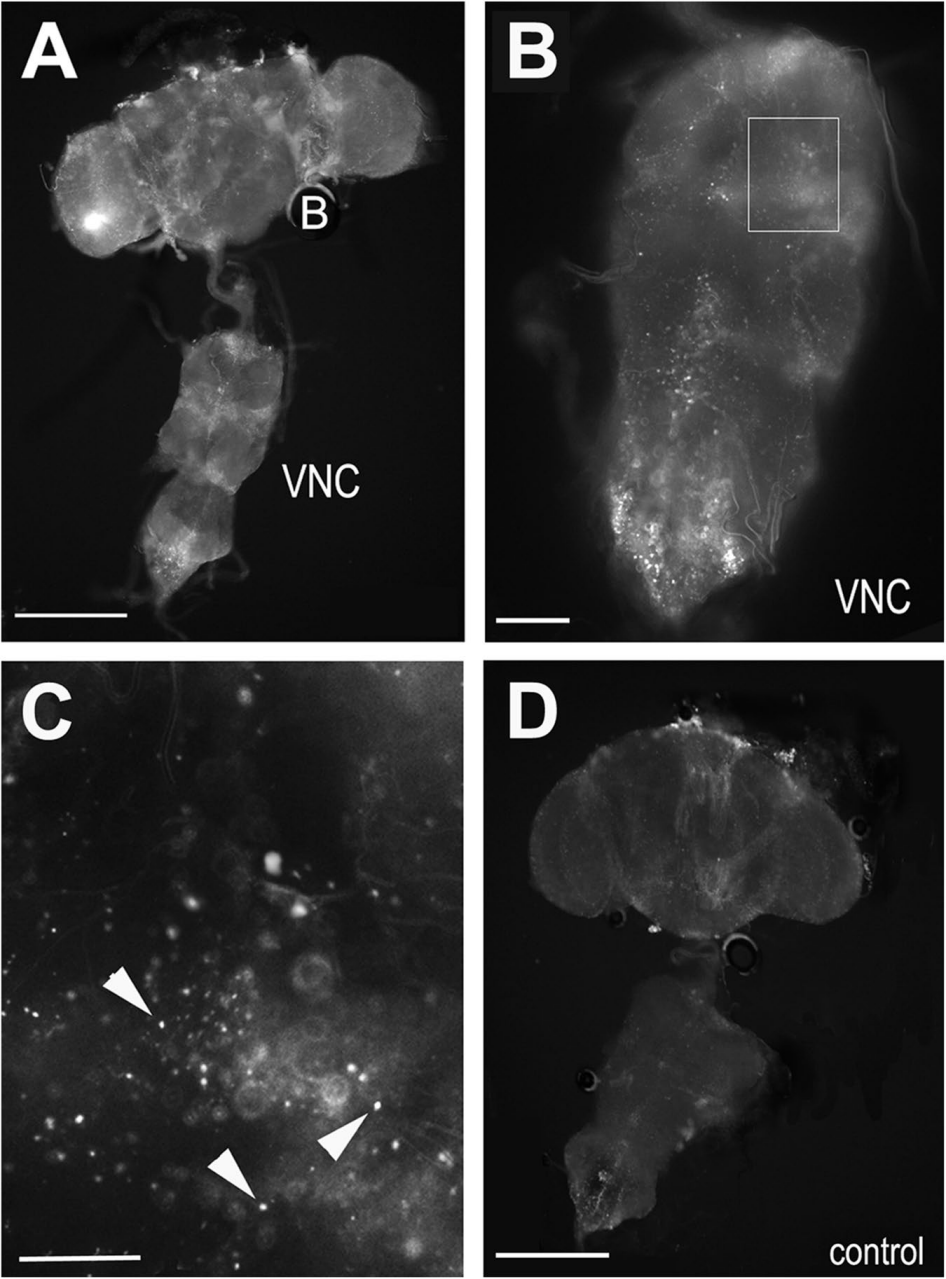
GFP reconstitution experiments show connections of the ICLIs to glutamatergic neurons in the ventral nerve cord. (**A**,**B**) Reconstituting GFP by expressing UAS-CD4-spGFP1-10 with *ntl*-GAL4 and lexAop-CD4-spGFP11 with GMR52D11 results in a punctuate staining in the brain (**B**) and ventral nerve cord (VCN). (**C**) Magnification of the area boxed in (**B**) shows reconstitution in puncta, possibly synapses (arrowheads). (**D**) A *ntl*-GAL4 > UAS-CD4-spGFP1-10 control fly does not show this pattern. Scale bars in A, D = 200 µm, in B = 50 µm, in C = 15 µm.

The ADLIs and ICLIs are well-suited to relay information from the sensory system and the brain to motor neurons, which are localized in the ventral nerve cord, thereby controlling behaviour. To determine whether these neurons do indeed connect to motor neurons, we used a GFP reconstitution system^26^. For this, UAS-CD4-spGFP1–10 was induced in the ICLIs and ADLIs with *ntl*-GAL4 while lexAop-CD4-spGFP11 was expressed with a *vGlut*-lexA driver, which due to motor neurons being glutamatergic is expressed in motor neurons^27^. As shown in Fig. 4A,B, we clearly detected GFP in the brain and ventral nerve cord in these flies, showing that these interneurons do form connections with glutamatergic neurons in the ventral nerve cord. A higher magnification revealed that GFP is detectable in a punctuate pattern along the midline of the ventral nerve cord, an area in which we also observed the *ntl*-GAL4 > DsRed and PKA-C3-positive varicosities (Fig. 4C, compare to Fig. 2E). In contrast, this was not the case in control flies that expressed only one part of GFP, UAS-CD4-spGFP1–10, via *ntl*-GAL4 (Fig. 4D). These findings supports our hypothesis that PKA-C3 is localized at synapses that connect the ADLIs and/or ICLIs with motor neurons.

## Discussion

As in other species, PKA complexes in *Drosophila* can contain different catalytic subunits but while the C1 subunit has been extensively studied, so far nothing was known about the specific distribution or function of the C3 subunit. Whereas the loss of PKA-C1 is lethal during development^3,28^, we show that the deletion of PKA-C3 is viable and exhibits no overt anatomical phenotypes. However, PKA-C3 mutants show behavioural deficits in mating, specifically a reduction in copulations. Due to their structural differences, we anticipated that PKA-C3 has specific functions not shared by PKA-C1 and indeed PKA-C1 expression could not compensate for the loss of PKA-C3. In addition, these two catalytic subunits appear to be present in different neuronal subpopulations in the adult brain. In agreement with its role in learning and memory, PKA-C1 is predominantly expressed in the mushroom bodies^29–31^ whereas we found PKA-C3 to be predominantly expressed in the ADLIs and ICLIs. That PKA-C3 is required in these interneurons was confirmed by our knockdown experiments which revealed that loss of PKA-C3 only in these four neurons is sufficient to induce the mating phenotypes. In addition, expression of PKA-C3 specifically in these neurons could completely rescue the copulation defects, showing that induction of PKA-C3 only in these neurons is sufficient for normal mating behaviour. Furthermore, using a constitutively inactive form of PKA-C3, we show that its function in copulation behaviour is indeed dependent on the kinase activity. Previous work by Jiang and colleagues demonstrated that the ICLIs and ADLIs play a critical role in mating behaviour and that successful mating requires Natalisin which is expressed in these neurons^25^. Because the ICLIs and ADLIs are present in our mutant, the mating defects are not caused by the loss of these neurons but by the loss of PKA-C3 in these neurons. Furthermore, using an antiserum against Natalisin, we could not detect changes in the levels or localization of Natalisin in the mutant, suggesting that PKA-C3 does not impair copulation via interfering with Natalisin signaling. Therefore, PKA-C3 seems to affect other pathways, however so far no targets of PKA-C3 are known. Identifying these targets could therefore provide more insights into the regulation of mating behaviour and the specific function of the ADLIs and ICLIs in this behaviour. We assume that PKA-C3 may be involved in the process of integrating information to achieve an appropriate motor neuron output. This is based on our findings that *Pka-C3*^*d*^ mutants can track the landmarks in the Buridan’s paradigm and can follow and court females, showing that they not only receive visual input but can also coordinate and execute locomotion output for these behaviours. The ADLIs and ICLIs connect the VLP, which receives visual, olfactory and gustatory input^32,33^, with the mushroom bodies, which are crucial for learning and memory^34^, and the ventral nerve cord. Because mutations that affect olfaction or taste show a reduction in the courtship index (CI)^35^ and the CI is not decreased in our mutant, we assume that none of the sensory inputs required for initiating and maintaining courtship is affected. However, it has been suggested that copulation also requires a feedback from the females^35^ and it is possible that *Pka-C3*^*d*^ mutants are unable to interpret or integrate these feedback responses to then initiate copulation. However, a better understanding of the mechanisms regulating these feedback responses is needed to address this issue and the possible role of PKA-C3 in this process.

## Methods

### Drosophila stocks

CantonS, *elav*-GAL4, UAS-*PKA-C1*, UAS-DsRed, GMR52D11-lexA and P{UAS-CD4-spGFP1–10}3, P{lexAop-CD4-spGFP11}3 were obtained from the Bloomington Stock Center. *Pka-C3*^*NIG*.*6117R*^ was obtained from the National Institute of Genetics (NIG-Fly), Japan. *natalisin*-GAL4 was kindly provided by Y. Park and Y-J. Kim and is described in^25^. *PBacf07226 and PBacPka-C3*^*f00695*^ were obtained from the Exelixis Collection at the Harvard Medical School. UAS-*PKA-C3* is described in^21^. The constitutively inactive PKA-C3 construct was generated by replacing aspartate 397 (D^397^, see Supp. Figure 3) with alanine by site–directed mutagenesis using the site-directed mutagenesis kit from clontech. D^397^ is part of the highly conserved YRDLKPEN core sequence of the catalytic loop and mutations in the conserved aspartate have been shown to abolish or dramatically reduce the catalytic activity^36–38^. The construct was tagged with HA. Flies were maintained on standard fly food under a 12:12 h light:dark cycle. Stocks were maintained at 18 °C while crosses and aging flies were maintained at 25 °C. The age of the flies in each experiment is indicated in the figures and figure legends.

### Immunohistochemistry

Brains were dissected in ice-cold PBS and transferred to 4% PFA in PBS. They were then fixed for 20 minutes at room temperature (RT) and washed four times with PBS/0.5% Triton (PBS-T) for 13 min each before blocking with 2% BSA in PBS-T for 2 hours at RT. Anti-RFP (Millipore AB3216) was used at 1:250 overnight at 4 C. Brains were then washed three times, 20 min each at RT and the secondary antibody applied (anti-rabbit-Cy2, Jackson ImmunoResearch) at 1:250 for 2 hours at RT. Brains were washed three times for 20 minutes with PBS-T and mounted in Glycergel for confocal imaging. For anti-PKA-C3 and Natalisin detection, brains were dissected in ice-cold PBS and fixed in 4% PFA for 45 min at room temperature. After washing four times for 13 min each with PBS-T, they were blocked with 2% BSA in PBS-T for 90 min with changing the blocking solution once in between. Anti-PKA-C3, raised against the DTKNFDDYPEKDWKPAK peptide in chicken (Aves Labs, Oregon), was used at 1:500 and rabbit anti-DmNTL4 (ref.^25^, kindly provided by Y-J. Kim, Gwangju Institute of Science and Technology, South Korea) at 1:1000 for 2 hours at RT, followed by incubation over night at 4 °C. Brains were then washed four times 13 min at RT and the secondary antibody (anti-chicken-Alexa488, Molecular Probes/Life Technologies) applied at 1:250 for 2 hours at RT. Brains were washed three times for 20 minutes with PBS-T and then mounted for confocal imaging using an Olympus FluoView 300 laser scanning confocal head mounted on an Olympus BX51 microscope.

### Buridan’s Paradigm

Flies with their wings cut (one day before testing) were analysed in a cylindrical, brightly illuminated arena that presented two opposing, inaccessible black stripes as described in^24^. Activity reflects the time spent walking during the 15-min observation time in %. The mean walking speed was averaged over the whole observation time in mm/s. GraphPad Prism and Shapiro-Wilk tests were used to test for normal distribution and unpaired t-tests for significance.

### Mating assays

Virgin wild type CantonS females were collected and kept on food vials for 3 days. Using an aspirator, single females were transferred to the courtship chamber (6 mm height × 10 mm diameter, kindly provided by C. Helfrich-Förster, University Würzburg) and an experimental male added that was also collected shortly after eclosion and aged for 3 days. Video recordings were done for 30 min using a Nikon Coolpix p500 camera. Successful mating occurred when the males copulated during the 30 minute recording period as described in^25^. The courtship assays were performed following the methods described in^39^. Chi square tests were used to determine significance. The Courtship Index (CI) was calculated by determining the time the males performed any type of courtship-associated behaviour divided by the total time of recording (10 min) as described in^40^. To determine a significant difference in the CI, GraphPad Prism and Mann-Whitney tests were used.

## Electronic supplementary material

Supplementary information
