# Supplementary material for: The PKA-C3 catalytic subunit is required in two pairs of interneurons for successful mating of Drosophila
Source: Sci Rep. 2018 Feb 6;8:2458. doi: 10.1038/s41598-018-20697-3 (PMC5802784; doi:10.1038/s41598-018-20697-3)
Supplement: Supplementary file 1 — Supplementary information [file 41598_2018_20697_MOESM1_ESM.pdf]

The PKA-C3 catalytic subunit is required in two pairs of interneurons for successful mating of *Drosophila*

Marlene Cassar[1], Elizabeth Sunderhaus[1], Jill S. Wentzell[1,3], Sara Kuntz[2], Roland Strauss[2], and Doris Kretzschmar[1*]

SUPPLEMENTARY INFORMATION

Methods:

RT-qPCR

RNA extraction from *Drosophila* heads and cDNA synthesis was performed as previously described in [1]. For quantitative PCR, the PerfeCTa FastMix II (Quantabio) was used and samples were analysed on a Bio-Rad iCycler iQ. We used CGAGCGGCCCAATTTGAATGT and AAACGGAAGCGGCAACACGA as primers for PKA-C3 and GGCGGCGAGAAGAAGATAGT and CTTGGCCTTGTCCTTGAAGT for the control reaction using the dTau gene. Fold changes were calculated using the Pfaffl method [2].

Western blots

Western Blots were performed as described in [3]. Lysates of 3 (for PKA-C3 overexpression) and 10 heads (for endogenous PKA-C3) were loaded on 8% SDS gels and blotted onto PROTRAN Nitrocellulose transfer membranes (Whatman). Anti-PKA-C3 was used at 1:1000 and anti-Tubulin or anti-Actin (1:50) was used as a loading control (obtained from the Developmental Studies Hybridoma Bank, DSHB, developed under the auspices of the NICHD and maintained by the Department of Biology, University of Iowa). Anti-HA (Covance) was used at 1:1000. Antibodies were diluted in TBST supplemented with 1% milk powder and incubated overnight at 4^0C. Bands were visualized using horseradish peroxidase-conjugated secondary antibodies (Jackson ImmunoResearch) at 1:1000 at RT for 2 hours and the SuperSignal West Pico chemiluminiscent substrate (ThermoScientific).

Immunohistochemistry

For the co-immunostainings in supplementary figures 5 and 6, anti-Bruchpilot (nc82, kindly provided by A. Hofbauer, University Regensburg, Germany) was used at 1.50. As

a secondary antibody, Cy5-labeled anti-mouse (Jackson ImmunoResearch) was used at 1:250.

PKA activity measurements

The PepTag® Assay for Non-Radioactive Detection of Protein Kinase C or cAMP-Dependent Protein Kinase kit from Promega was used for PKA activity measurement. Five fly heads were homogenized in 50µl extraction buffer and centrifuged at 14,000xg for five minutes. 2 µl of the supernatant was immediately used for activity measurement assays for each sample, while 20µl were used to perform Bradford assays [4]. Activity measurements were performed using 0.04µg/µl PepTag A1 peptide, and 250mM isobutylmethylxanthine (IBMX) with cAMP (0.4 mM) added to the reaction. The reaction was performed according to the kit protocol and evaluated by luminosity measurement of the gel picture by determining the p/q quotient of the luminosity value of phosphorylated to unphosphorylated kemptide peptide. Values were normalized per ng amount of protein in the lysate, as determined by the Bradford assays. As positive control, we used 2µg/ml PKA provided by the kit, negative controls contained water instead of fly head lysate. Incubation time was 30min.

References:

1 Cassar, M. *et al.* A dopamine receptor contributes to paraquat-induced neurotoxicity in Drosophila. *Hum Mol Genet* **24**, 197-212, doi:10.1093/hmg/ddu430 (2015).

2 Pfaffl, M. W. A new mathematical model for relative quantification in real-time RT-PCR. *Nucleic acids research* **29**, e45 (2001).

3 Carmine-Simmen, K. *et al.* Neurotoxic effects induced by the Drosophila amyloid-beta peptide suggest a conserved toxic function. *Neurobiol Dis* **33**, 274-281, doi:10.1016/j.nbd.2008.10.014 (2009).

4 Bradford, M. M. A rapid and sensitive method for the quantitation of microgram quantities of protein utilizing the principle of protein-dye binding. *Anal Biochem* **72**, 248-254 (1976).

Figure legends:

Supplementary figure 1

Reduced levels of PKA-C3 in the RNAi knockdown. Expressing the Pka-C3$^{NIG.6117R}$ RNAi construct with *Appl*-GAL4 results in a significant reduction of relative *Pka-C3* mRNA levels compared to the controls expressing only the *Appl*-GAL4 driver (set to 1) or only the RNAi construct. Three independent experiments were performed. SEM is indicated. ** p<0.01.

Supplementary figure 2

Generating the deletion in PKA-C3. (**A**) Genomic region of *Pka-C3* showing the intron-exon structure of the two alternative *Pka-C3* mRNA transcripts. The position of the two insertion lines PBacPka-C3^{f00695} and PBacf07226 that were used for a FLP/FRT-mediated recombination is indicated by the light blue arrowheads and the created deletion by the light blue line. (**B**) Western blot using the PKA-C3 antiserum and head homogenates. Three bands around 60kD, the predicted size of the two PKA-C3 isoforms is 57kD and 65kD, are detectable in wild-type (WT) flies. The levels of the protein corresponding to the band in the middle (arrow) are strongly increased in flies expressing additional PKA-C3 pan-neuronally with *Appl*-GAL4, validating that this band corresponds to PKA-C3. The PKA-C3 band is missing in *Pka-C3^{d}* flies whereas the unspecific bands are still present. A loading control using anti-Tubulin is shown below. The Western blot was performed with several putative recombination lines, the part indicated by the box is shown in the magnification.

Supplementary figure 3

Loss of PKA-C3 does not affect courtship. Measuring the Courtship Index, by determining the time the males engaged in any of the steps of courtship behaviour like tapping or wing vibration, we did not detect a difference between controls (wild type CS) and *Pka-C3^{d}* males. The number of tested males and the SEM are indicated.

Supplementary figure 4

Alignment of the three *Drosophila* PKA catalytic subunits. The peptide used for the generation of the antiserum is highlighted in blue, the catalytic loop in green, and the D to A mutation used to create the constitutive inactive PKA-C3 is shown in red below.

Supplementary figure 5

(**A**) Expressing dsRed with *ntl*-GAL4 shows staining in the ADLIs and ICLIs (arrowheads). (**B**) Overlay of the dsRed and PKA-C3 staining shows co-localization of PKA-C3 and dsRed. (**C**) Overlay of immunostainings of anti-dsRed, anti-PKA-C3, and anti-Bruchpilot (nc82) shows the localization of the ADLIs and ICLIs in the brain.

Supplementary figure 6

(**A**) Immunohistochemistry with anti-PKA-C3 on a brain whole-mount from a *Appl*-GAL4> Pka-C3$^{NIG.6117R}$ knock-down fly shows unspecific staining in the MBs whereas the expression in the ADLIs and ICLIs (arrowheads) is strongly reduced. (**B**) Using an antiserum against Natalisin, we can easily detected the ADLIs and ICLIs in the knockdown. (**C**) Overlay of the anti-PKA-C3 and anti-Natalisin staining. (D) Triple staining with anti-PKA-C3, anti-Natalisin, and anti-Bruchpilot (nc82).

Supplementary figure 7

(**A**) Western blot showing higher expression levels of the PKA-C3^{D397A} (PKA-C3^{CI}) than the wild type PKA-C3 constructs. Both were tagged with HA and detected using anti-HA. A loading control with anti-Actin is shown below. (**B**) PKA activity measurements show an increase in activity compared to the control when wild-type PKA is expressed but not when PKA-C3^{D397A} is expressed. Three independent experiments were done with duplicate measurements each. The SEMs are indicated. * $p<0.05$, ns not significant.

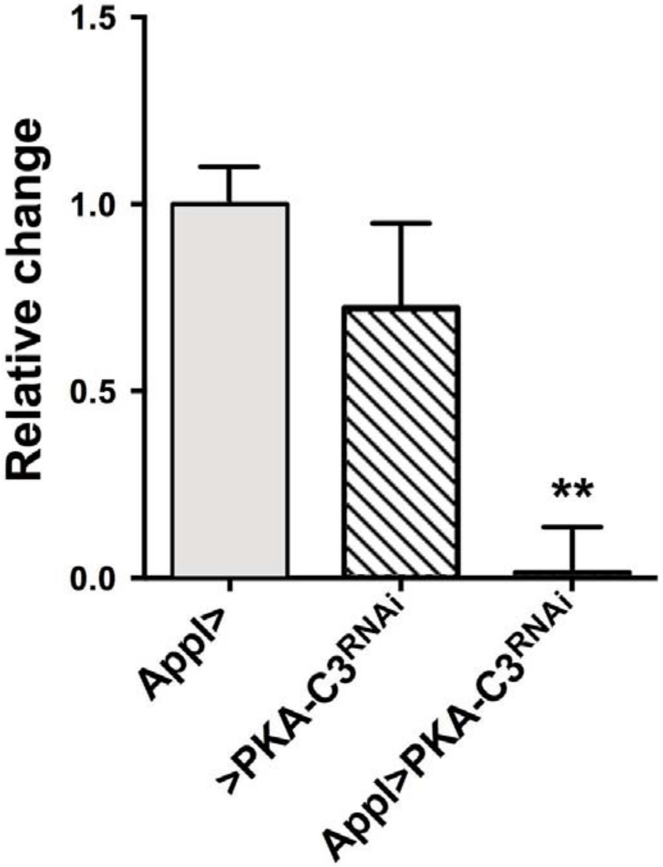

1.5
1.0
0.5
0.0
Relative change
**
Appl>
>PKA-C3RNAi
Appl>PKA-C3RNAi

A

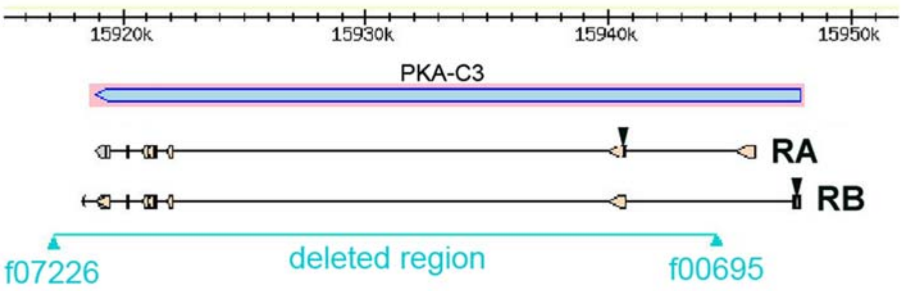
15920k
15930k
15940k
15950k
PKA-C3
RA
RB
f07226
deleted region
f00695

B

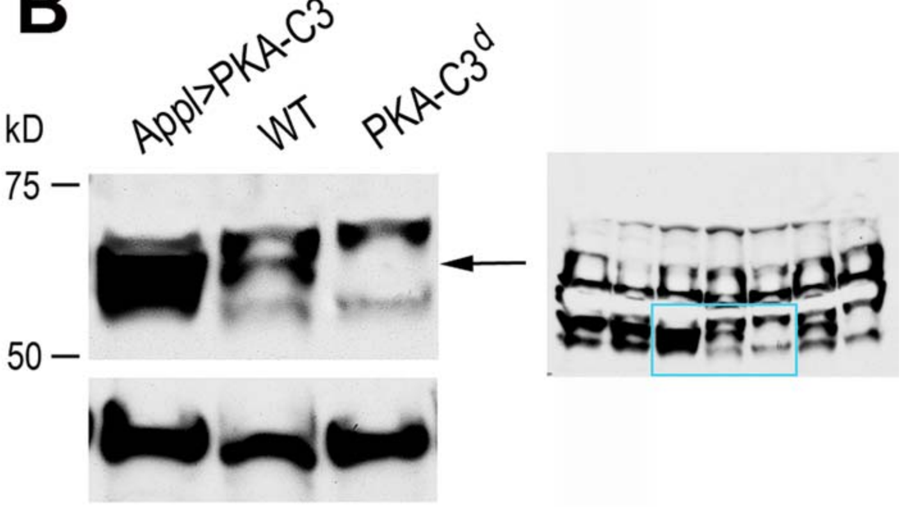
kD
Appl>PKA-C3
WT
PKA-C3d
75
50

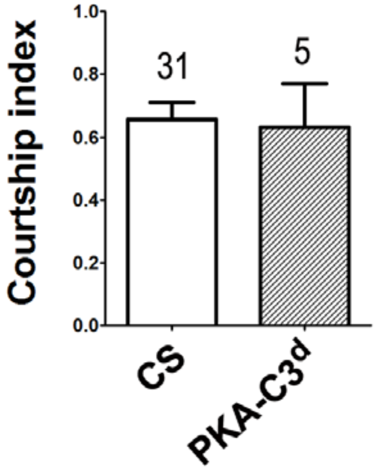

Courtship index
1.0
0.8
0.6
0.4
0.2
0.0
31
5
CS
PKA-C3d

```
PKA-C2          ------------------------------------------------------------
PKA-C1          ------------------------------------------------------------
PKA-C3          MSTATCARFCTPLSSGTAGSTSKLTTGNGSGNTMTSAYKIPSNNSTTANDSSNTETTFTF

PKA-C2          ------------------------------------------------------------
PKA-C1          ------------------------------------------------------------
PKA-C3          KLGRSNGRSSSNVASSESSDPLESDYSEEDPEQEQQRPDPATNSRSSSTATTTTTSSADH

PKA-C2          ----------------------------MSQHTSQYVFNSKEDYNVILDNMSREFEERWNH
PKA-C1          ----------------MGNNATTSNKKVDAAETVKEF---------LEQAKEEFEDKWRR
PKA-C3          DNDVDEEDEEDDENEGEGNGRDADDATHDSSESIEEDDGNETDDEEDDDESEESSSVQTA
                                            .   :              :: ..* ..

PKA-C2          QTQSPYTNLENYITRAVLGNGSFGTVMLVREKSGKNYYAAKMMSKEDLVRLKQVAHVHNE
PKA-C1          NPTNT-AALDDFERIKTLGTGSFGRVMIVQHKPTKDYYAMKILDKQKVVKLKQVEHTLNE
PKA-C3          KGVRK-YHLDDYQIIKTVGTGTFGRVCLCRDRISEKYCAMKILAMTEVIRLKQIEHVKNE
                :       *:::    .:*.*:** * : :.:  :.* * *::   .:::***: *. **

PKA-C2          KHVLNAARFPFLIYLVDSTKCFDYLYLILPLVNGGELFSYHRRVRKFNEKHARFYAAQVA
PKA-C1          KRILQAIQFPFLVSLRYHFKDNSNLYMVLEYVPGGEMFSHLRKVGRFSEPHSRFYAAQIV
PKA-C3          RNILREIRHPFVISLEWSTKDDSNLYMIFDYVCGGELFTYLRNAGKFTSQTSNFYAAEIV
                :.:*.  :.**:: *    *  . **:::  * ***:*:: *.. :*..  :.****::.

PKA-C2          LALEYMHKMHLMYRDLKPENILLDQRGYIKITDFGFTKRVDGRTSTLCGTPEYLAPEIVQ
PKA-C1          LAFEYLHYLDLIYRDLKPENLLIDSQGYLKVTDFGFAKRVKGRTWTLCGTPEYLAPEIIL
PKA-C3          SALEYLHSLQIVYRDLKPENLLINRDGHLKITDFGFAKKLRDRTWTLCGTPEYIAPEIIQ
                 *:**:* :.::********:*::  *::*:*****:*:: .** ********:****:
                             D>A
PKA-C2          LRPYNKSVDWWAFGILVYEFVAGRSPFAIHNRDVILMYSKICICDYKMPSYFTSQLRSLV
PKA-C1          SKGYNKAVDWWALGVLVYEMAAGYPPFFAD--QPIQIYEKIVSGKVRFPSHFGSDLKDLL
PKA-C3          SKGHNKAVDWWALGVLIYEMLVGYPPFYDE--QPFGIYEKILSGKIEWERHMDPIAKDLI
                 : :**:*****:*:*:**: .*  **  .  : : :*.**   . .   ::    :.*:

PKA-C2          ESLMQVDTSKRLGNSNDGSSDVKSHPWFQGVDWFGILNQEVTAPYQPTISGAEDLSNFEN
PKA-C1          RNLLQVDLTKRYGNLKAGVNDIKNQKWFASTDWIAIFQKKIEAPFIPRCKGPGDTSNFDD
PKA-C3          KKLLVNDRTKRLGNMKNGADDVKRHRWFKHLNWNDVYSKKLKPPILPDVHHDGDTKNFDD
                ..*:  * :** ** : * .*:* : **   :*  : .:::  *  *      * .**::

PKA-C2          FEFKDRYKSRIN--RHPELFANF
PKA-C1          YEEEALRISSTEK--CAKEFAEF
PKA-C3          YPEKDWKPAKAVDQRDLQYFNDF
                :  :    :        : * :*
```

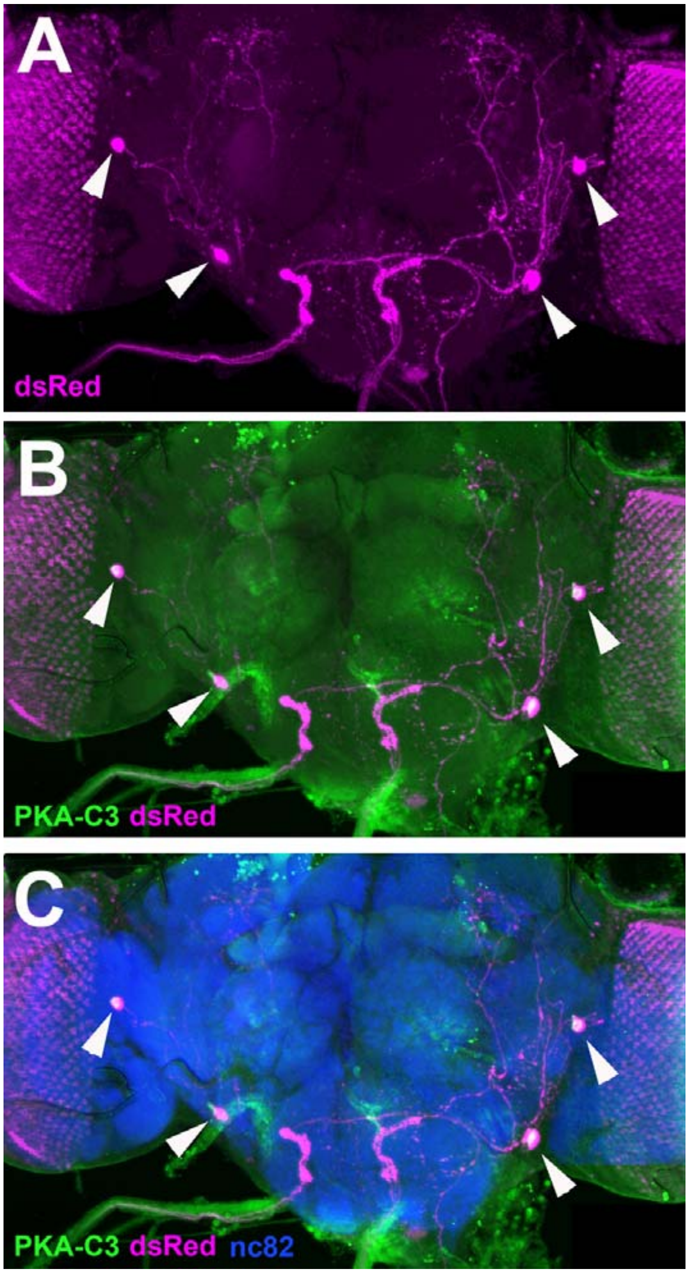
A
dsRed
B
PKA-C3 dsRed
C
PKA-C3 dsRed nc82

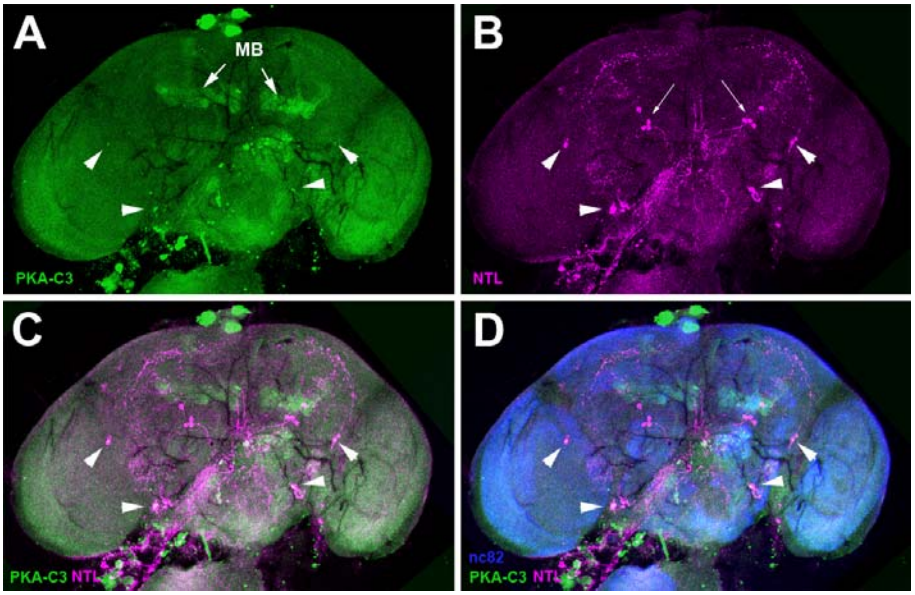
A
MB
PKA-C3
B
NTL
C
PKA-C3 NTL
D
nc82
PKA-C3 NTL

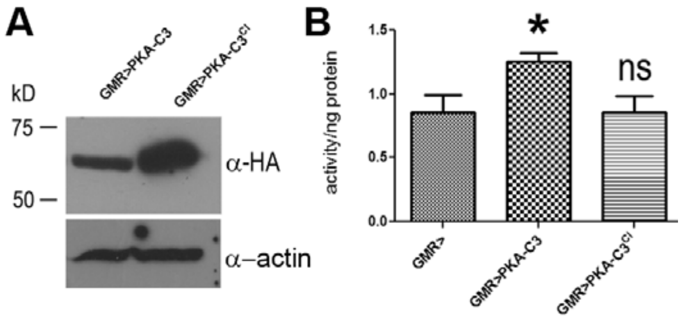
A
GMR>PKA-C3
GMR>PKA-C3^CI
kD
75
50
α-HA
α–actin
B
*
ns
1.5
1.0
0.5
0.0
activity/ng protein
GMR>
GMR>PKA-C3
GMR>PKA-C3^CI
